# The magnitude of *Schistosoma mansoni* and its associated risk factors among Sebatamit primary school children, rural Bahir Dar, Northwest Ethiopia: a cross-sectional study

**DOI:** 10.1186/s13104-019-4498-3

**Published:** 2019-07-22

**Authors:** Lemma Workineh, Mulat Yimer, Woyneshet Gelaye, Desalegn Muleta

**Affiliations:** 1 Department of Medical Laboratory Sciences, College of Medicine and Health Sciences, Debre Tabor University, Debre Tabor, Ethiopia; 2 Department of Medical Laboratory Sciences, College of Medicine and Health Sciences, Bahir Dar University, Bahir Dar, Ethiopia; 3 Department of Medical Laboratory Sciences, College of Medicine and Health Sciences, MizanTepi University, Mizan Teferi, Ethiopia

**Keywords:** Rural Bahir dar, Sebatamit primary school, Schistosoma *mansoni*

## Abstract

**Objective:**

The aim of this study was to determine the magnitude of *Schistosoma mansoni* and its associated risk factors in the study area.

**Results:**

Of 422 school children, 223 (52.8%) and 199 (47.2%) were males and females, respectively. The Overall prevalence of *Schistosoma mansoni* infection was 24.9% (105/422). Seventy-five out of 422 (71.4%) of the infected individuals showed light infections. The overall mean intensity of *Schistosoma mansoni* in the study was 106.16 eggs per gram of stool. Age (p = 0.013), swimming habit (p = 0.001), participating in irrigational activities (p = 0.03) and washing clothes in the river (p = 0.039) were factors associated with *Schistosoma mansoni* infection.

## Introduction

Schistosomiasis is a disease caused by trematode worms of the genus *Schistosoma* [1]. The three medically important *Schistosoma* species (*Schistosoma* spp.) are *Schistosoma mansoni* (*S. mansoni*), *Schistosoma hematobium* (*S. hematobium*) and *Schistosoma japonicum* (*S. japonium*) [1]. Among these, *S. hematobium* is the cause of urinary schistosomiasis and the other two cause intestinal schistosomiasis [2]. It is one of the most prevalent neglected tropical diseases (NTDs) and a major public health problem in 77 developing countries in tropics and subtropics [3]. Over 240 million people are infected and 700 million people are at risk of schistosomiasis worldwide [4].

In Africa, schistosomiasis due to *S. mansoni* and *S. haematobium* are the second most common NTDs after hookworm infection [5]. In sub-Saharan Africa, over 3000,000 deaths occur annually due to schistosomiasis [6]. In Ethiopia, 37.3 million people are living in schistosomiasis endemic areas, comprising 3.4 million pre-school children, 12.3 million school-aged children, and 21.6 million adults [7]. As a result, the magnitude and impact of schistosomiasis infection is high in Ethiopia even though several control methods have been conducted.

In North West Ethiopia, different data have indicated that there is high prevalence of *S. mansoni* in the region including 89.9% in Sanja General elementary school [8], 82.8% in Sanja and Ewukat Amba primary school [9], 56.6% in Tach Armachio district [10], 37% in Zarima town of Gonder [11], 20.6% in Gorgora town [12], 14.3% in Ethiopian Orthodox church students around Lake Tana [13] and 2.8% in Bahir Dar Shimbit elementary school [14].

In our study area, the school children are at risk for *S. mansoni* due to their contact to Abay and Andassa Rivers for their daily activities like swimming, fetching, washing clothes and agricultural activities like irrigation. Therefore the objective of this study was to determine the magnitude of *S. mansoni* in and its associated factors Sebatamit primary school children.

## Main text

### Methods and materials

#### Study design and area

A school-based cross-sectional study was conducted at Sebatamit primary school from April to May, 2018. The school is located 7 km from Bahir Dar city which is the capital city of Amhara Regional State. The altitude of the area is 1820 m above sea level. The annual temperature of the area is 11–29 °C. The residents at the study area use Abbay and Andassa Rivers for recreational, irrigation, drinking and cooking purposes.

#### Sample size determination and sampling technique

Sample size was determined using 50% prevalence since there was no study conducted regarding this topic in the area, 95% CI, 5% margin of error with 10% non-response rate; hence using single proportion formula, 422 students were included in the study. The total number of children from grade 1–8 in the school was 1734. There were four sections in each grade. Then, proportional allocation formula was used to determine to the number of children sampled from each grade. Hence, the numbers of students sampled from grade 1–8, respectively were: 65, 62, 58, 55, 59, 43, 46 and 34 having a total of 422 children. Then, by using class rosters as a sampling frame, systematic sampling technique was used to select the sections from each grade and the school children from the selected sections.

#### Stool sample collection for parasitological examination

Each of the school children was informed to provide total of 2 g of stool sample. Then, the stool samples were preserved with 10% formalin and transported to Bahir Dar University Microbiology and Parasitology teaching Laboratory for Kato-Katz. Moreover, standardized questionnaires were used to collect data on socio-demographic characteristics of the study participants and associated risk factors of *S. mansoni* infection.

#### Parasitological stool examination by Kato Katz technique

For Kato-Katz technique, two Kato slides were prepared for every school children’s stool sample using fixed quantity of sieved 41.7 mg of stool on a punched template [15]. They were then mounted on slides and covered with malachite green impregnated cellophane. The slides were observed within 1 h under the microscope at a magnification of 10× objective. The mean total number of eggs was expressed as eggs per gram (EPG) of stool.

#### Quality control

Ten percent of the examined Kato-Katz slides were randomly selected and re-examined at the end by qualified laboratory technologist who were blind for the first result. Finally, all results were documented on log book by its unique ID number and copied to the questionnaire by principal investigators.

#### Data analysis

Data were analyzed using SPSS version 23. The associated risk factors for S*. mansoni* infection were first analyzed by bivariate logistic regression. Then, to control the possible confounding factors, variables with p value < 0.2 were adjusted by multivariate logistic by stepwise variable selection. Finally, variables with p value < 0.05 were considered as statistically significant.

#### Ethical considerations

Ethical clearance was obtained from Bahir Dar University College of Medicine and Health Sciences institutional review board (IRB) and approved with protocol number 070/18-04 on 04 April 2018 prior to start the study. Permission letter was obtained from Bahir Dar city education department and Sebatamit primary school director office. Informed written consent was obtained from the children’s parents and assent was obtained from the children. Those school children who were positive for *S. mansoni* were treated accordingly.

### Results

#### Sociodemographic characteristics of the study participants

Out of 422 school children, 223 (52.8%) and 199 (47.2%) were males and were females, respectively. The age of the school children ranged from 7 to 18 years with the mean age of 11.5 year. Two hundred sixty out of 422 (53.6%), 150/422 (35.5%) and 46/422 (10.9%) of the school children were from 11 to 14, 7 to 10 and 15 to 18 years age group, respectively. Most of the school children were from grades 1–4 accounted 240/422 (56.9%) followed by 182/422 (43.1%) from grades 5–8.

#### The prevalence of *S. mansoni* infection

One hundred five children were found to be positive to *S. mansoni* infection giving an overall prevalence of 24.9%. The males were found to be more infected than the females with 66 (15.6%) of the males being infected. The age group with the highest prevalence value for *S. mansoni* infection was between 11 and 14 years with 66 (15.6%) of them found to be infected while the least prevalence of 1.4% was among 6 children in the age group 15–18 years as shown in Table 1.

**Table 1:** *Schistosoma mansoni* infections across sociodemographic factors among school children at Sebatamit primary school from April to May, 2018

Characteristics	*S. mansoni* infection		
	Yes N (%)	No N (%)	Total N (%)
Sex			
Male	66 (15.6)	157 (37.2)	223 (52.8)
Female	39 (9.2)	160 (37.9)	199 (47.2)
Total	105 (24.9)	317 (75.1)	422 (100)
Age			
7–10	33 (7.8)	117 (27.7)	150 (35.5)
11–14	66 (15.6)	160 (37.9)	226 (53.6)
15–18	6 (1.4)	40 (9.4)	46 (10.9)
Total	105 (24.9)	317 (75.1)	422 (100)
Grade			
1–4	65 (15.4)	175 (41.4)	240 (56.90
5–8	40 (9.4)	142 (33.6)	182 (43.1)
Total	105 (24.9)	317 (75.1)	422 (100)
Father educational status			
Illiterate	57 (13.50	172 (40.7)	229 (54.3)
Literate	48 (11.4)	145 (34.3)	193 (45.7)
Total	105 (24.9)	317 (75.1)	422 (100)
Mother educational status			
Illiterate	82 (19.4)	244 (57.8)	326 (77.3)
Literate	23 (5.4)	73 (17.2)	96 (22.7)
Total	105 (24.9)	317 (75.1)	422 (100)

#### Intensity of *S. mansoni* infection

Out of 105 school children, 75 (71.4%), 25 (23.8%) and 5 (4.8%) had light, moderate and heavy infections, respectively according to WHO classification [15]. Thirty (76.6%), 7 (17.9%) and 2 (5.2%) of female students had light, moderate and heavy infections, respectively where as 45 (68.2%), 18 (27.3%) and 3 (4.5%) of male students had light, moderate and heavy infections, respectively. The age group with the highest light infections was between 11 and 14 years with 48 (72.7%) while the least was between 15 and 18 years with 4 (66.8%). Both 7–10 and 11–14 years had similar moderate infections with 8 (24.2%) and 16 (24.2%), respectively while the highest heavy infections, 1 (16.6%) was found in 15–18 years.

#### Analysis of risk factors associated with *S. mansoni* infection

Among the potential associated risk factors of *S. mansoni* infection, swimming habit (p = 0.001), irrigational activities (p = 0.005) and washing clothes in the river (p = 0.045) were statistically significant with *S. mansoni* infection in bivariate logistic regression analysis. However, factors like crossing the river on bare foot and open defecation were not statistically significant (p > 0.05). From sociodemographic factors, sex of the children (p = 0.018) and age of the children (p = 0.046) were statistically associated in bivariate logistic regression analysis. But academic level of the students was not statistically significant (p = 0.23) as shown in Table 2.

**Table 2:** Bivariate logistic regression analysis of associated factors of *S. mansoni* among Sebatamit primary school children, from April to May, 2018

Associated factors	*S. mansoni* infections		COR (95% CI)	p value
	Positive	Negative		
Sex				
Male	66	157	1.725 (1.096–2.713)	0.018
Female	39	160	1	
Age				
7–10	33	117	1.880 (0.734–4.819)	0.188
11–14	66	160	2.750 (1.113–6.796)	0.028
15–18	6	40	1	
Grade				
1–4	65	175	1.319 (0.839–2.702)	0.23
5–8	40	142	1	
Swimming habit				
Yes	79	160	2.981 (1.818–4.890)	0.001
No	26	157	1	
Irrigational activities				
Yes	57	108	1.899 (1.214–2.970)	0.005
No	48	209	1	
Crossing the river with bare foot				
Yes	49	127	1.309 (0.839–2.014)	0.235
No	56	190	1	
Washing clothes in the river				
Yes	78	196	1.783 (1.090–2.919)	0.021
No	27	121	1	
Open defecation				
Yes	46	161	0.755 (0.485–1.187)	0.216
No	59	156	1	

All bivariate results that had p-value < 0.2 were subjected to multivariate logistic regression model to control possible confounding factors as shown in Table 2. After adjustment to multivariate logistic regression model, sex of the school children (p = 0.659) was not statistically significant. The school children from 11 to 14 years were 3.2 more likely to be infected with *S. mansoni* than those who were from 15 to 18 years (AOR = 3.2, 95% CI 1.274–8.151, p = 0.013). The odds of being infected by *S. mansoni* among school children who swam was 2.8 more likely as compared to those who did not (AOR = 2.8, 95% CI 1.702–4.699, p = 0.001). The school children who participated in irrigational activities were 1.675 more likely to be infected with *S. mansoni* than those who did not (AOR = 1.675, 95% CI 1.050–2.671, p = 0.030). The school children who washed their clothes in the river were 1.711 more likely to be infected with *S. mansoni* than those who didn’t (AOR = 0.1.711, 95% CI 1.028–2.851, p = 0.039) as shown in Table 3.

**Table 3:** Multivariate logistic regression analysis of associated factors of *S. mansoni* among Sebatamit primary school children from April to May, 2018

Associated factors	*S. mansoni* infection		AOR (95% CI)	p value
	Positive	Negative		
Sex				
Male	66	157	1.129 (0.675–1.862)	0.659
Female	39	160	1	
Age				
7–10	33	117	2.278 (0.867–4.5.985)	0.095
11–14	66	160	3.223 (1.274–8.151)	0.013
15–18	6	40	1	
Swimming habit				
Yes	79	160	2.828 (1.702–4.699)	0.001
No	26	157	1	
Irrigational activities				
Yes	57	108	1.675 (1.050–2.671)	0.030
No	48	209	1	
Washing clothes nearby river				
Yes	76	195	1.711 (1.028–2.851)	0.039
No	29	122	1	

*AOR* adjusted odds ratio

### Discussions

In this study, the overall prevalence of *S. mansoni* infection was 24.9%. This prevalence was slightly in with other studies such as 24% in Jimma [16], 23.9% in Mekelle [17], 26.5% in Democratic Republic of Congo [18]. However, the prevalence of this study found to be lower compared to other findings in different parts of Ethiopia; including 73.7% in Bushulo village of Southern Ethiopia [19], 31% along lake Hawassa of Southern Ethiopia [20], 58.6% in Wolaita Zone [21], 67.6% in Horo Guduru Wollega [22], 73.9% and 42.4% in Tigray [23, 24], 37% in Zarima town of Gondar [11], 89.9% in Sanja General elementary school of Gondar [8], 82.8% in Sanja and Ewukat Amba primary school Gondar of [9], 56.6% in Tach Armachiho district of Gonde [10], 60.5% in Kenya [25], 84.01% in Tanzania [26].

The variations in the prevalence in this study might be due to some factors such as: the presence of fast running rivers (Abay and Andassa) in the area leading to low availability of vector snails that are mainly found to prefer stagnant or slow-moving water bodies [27]; the sociodemographic and other factors (immune status); the distance of the study area to the source of water may be a major cause of variation in prevalence; sample size..

On the other hand, the prevalence of this study was higher than 119 (20.6%) in Gorgora town [12], 14.3% in Ethiopian Orthodox church students around Lake Tana [13] and 2.8%) in Bahir Dar Shimbit elementary school [14]. The reason for these variations might be due to poor sanitation; in our study area greater than 50% of the study participants had no latrine in their home.

In our study, most of infections were light infections. This is in line with the study done in Jimma zone [16] and Mekelle city [17]. However, the highest moderate infections were observed in Sanja town [8, 9] and in Gorgora town [12] whereas in Wolayita zone [21] the highest heavy infections were observed. The difference in infection intensity could have been due to the frequency of exposure to contaminated water, the burden of the adult worms in hosts and immune status of the study participants.

In this study regarding the associated factors to *S. mansoni* infection, sex as an associated factor to *S. mansoni* infection was not retained on the multivariate analysis though it was significant in bivariate analysis which was similar to the findings from different parts of Ethiopia [8, 9 and 22]. Contrasting findings on *S. mansoni* infection among males and females were identified. Higher prevalence of *S. mansoni* infection among males was reported in Jimma Zone [16] and Wolayita Zone [21], whereas the study conducted in Gorgora and Mekelle [12, 17] reported the opposite findings (higher prevalence in females than males). These differences might be due to the difference in the exposure status of both sexes to cercarial contaminated water source.

The school children who were from 11 to 14 years were 3.2 more likely to be infected with *S. mansoni* infection than those in 15–18 years. This finding is in line with the study done in Gorgora [12], Jimma zone [16], Mekelle [17] and Kenya [25]. This could be due to higher rate of water contact activities for recreational purposes and for bathing by the school children within 11–14 years and the concomitant immunity to re-infection in school children aged 15–18 years. Swimming habit, participation in irrigational activities and washing clothes in the river were factors associated with *S. mansoni* infection.

### Conclusion

The overall prevalence of *S. mansoni* infection in the present study was 24.9%. Majority of the infections were light infection with mean intensity of 106.6 EPG. Age; swimming habit, irrigational activities, washing clothes in the river were the factors associated with *S. mansoni* infection. However, deworming program and community awareness should be strengthened to control *S. mansoni* infection.

## Limitation

We did not include associated factors and sociodemographic factors like awareness of the study. Participants about schistosomiasis, occupational status of parents, and religion of the study participants and residence of the participants.

## Data Availability

All data and material were obtained from Google Scholar, Pub Med and Med Line data bases.

## Abbreviations

AOR: adjusted odds ratio
COR: crude odds ratio
EPG: eggs per gram of stool
IRB: institutional review board
NTDs: neglected tropical diseases
